# A Pragmatic and Systemic Approach to Advance Research in Health Policy and Management: Comment on "Insights Gained From a Re-analysis of Five Improvement Cases in Healthcare Integrating System Dynamics Into Action Research"

**DOI:** 10.34172/ijhpm.2022.7690

**Published:** 2022-11-27

**Authors:** Guido Noto

**Affiliations:** Department of Economics, University of Messina, Messina, Italy.

**Keywords:** Action Research, System Dynamics, Healthcare, Pragmatism, Management

## Abstract

"Healthcare is complex" - or similar sentences – is a statement that introduces a wide number of scientific articles dealing with health policy and management issues. We all agree that healthcare is complex, but most studies, although using this kind of sentence to introduce their background, do little to effectively deal with such complexity in their analyses. Holmström et al proposed a methodological approach to tackle healthcare complexity by integrating system dynamics (SD) into action research (AR). This commentary highlights three touch points that makes the combination of AR and SD feasible, namely the epistemological ground, the use of experimentation and the collaborative approach. The proposed approach addresses some of the key sources of the complexity characterizing healthcare settings.

## Background

 Healthcare is complex, and we may all agree with such a statement. However, in modern science, there is little universal agreement on how to define and frame complexity.^1^ The key features that makes healthcare complex could be grouped into three elements, namely pluralism, institutional fragmentation and uncertainty. These characteristics defines what literature calls ‘wickedness,’ ie, a set of conditions that prevent an agreement on problems definition and the related solutions.^2^ To cope with such wickedness, the adoption of a collaborative and systemic view of problems is desirable.^2^

 The intuition beyond the paper of Holmström and colleagues^3^ to cope with complexity in the healthcare sector is based on the integration of two methodological approaches that both allow coping with the above-mentioned sources of complexity. In particular, Holmström et al,^3^ suggest integrating system dynamics (SD) into action research (AR) strategies to conduct research in the healthcare policy and management realm (and, perhaps, also in other social systems).

 The term AR was first coined by Lewin^4^ who defined it as “a comparative research on the conditions and effects of various forms of social action and research leading to social action.” AR was proposed so as to cope with the perception that traditional science was not helping in the resolution of critical and complex social problems. In AR, practitioners and social scientists experiment on social systems to find ways to bring about needed changes. This research strategy is thus aimed at contributing to both the theoretical domain – ie, etic – and to the practical domain – ie, emic – closing the gap between theory and practice often characterizing social sciences research^5^ and natural ones.^6^ Applications of AR thus responded to the inability of traditional reductionist approaches to grasp and take into account the complexity of certain social systems. This explains why AR has been largely applied in the health policy and management domain.

 Being AR a pragmatic research methodology, there are no specific indications on how interventions and changes should be put in place in order to experiment on the social system analysed. What is given is the process, which is conceived as “a spiral of steps, each of which is composed of a circle of planning, action, and fact-finding about the result of the action.”^4^ In this sense, AR gives floor to the integration of various research techniques and approaches that may support experimentation.

 SD is a methodological approach developed by Jay Wright Forrester for modelling and simulating complex physical and social systems and experimenting with the models to design strategies for management and change.^7,8^ Thanks to the systemic and dynamic view intrinsic to this technique, SD has been widely applied to deal with complex health policy and management problems.^9^

 This commentary critically analyses the integration of SD into AR – proposed by Holmström et al^3^ – as an approach to cope with the complexity characterizing policy and management improvements in healthcare.

## Action Research and System Dynamics Touch Points

 From Holmström et al^3^ integration of SD into AR in healthcare, it is possible two identify three key touch points that allow the integration of the two approaches. These are: the epistemological ground; the use of experimentation; and the collaborative approach to research.

###  Epistemological Ground

 One of the reasons why AR and SD can be successfully integrated in healthcare studies is identified in the epistemological ground that both share.

 AR can be defined as a research strategy,^10^ while we refer to SD as a research technique, ie, a tool to collect and analyse data and information in order to produce the research output. As such, while AR can be carried out through the support of other research techniques, SD can also support other research strategies (eg, case study, experiment, grounded theory). What makes the integration of SD into AR feasible, favourable and even desirable, is that both fit well within the pragmatism research philosophy.^11,12^ Pragmatism focuses on the practical understanding of real-world issues. According to this philosophy, research strategies, methods and techniques should be chosen in order to provide useful answers to practical problems – as represented in the research questions.^10^ Pragmatism may also be considered an adequate epistemological ground to host studies and researches dealing with complexity and with complementarity – according to which no single research technique, theoretical position, or modelling strategy provides a complete account of all system’s features – as it allows to acknowledge multiple viewpoints and distinct levels of explanation of healthcare dynamics.^13^

 The five improvement cases reported by Holmström and colleagues^3^ are all referred to healthcare organizational improvement processes and learning. As both AR and SD have been widely used to address research objectives in this domain (in particular, organizational learning), the suggested approach fits well in order to tackle Holmström et al^3^ research aim.

###  Experimentation

 Another feature that clearly emerged from the article of Holmström et al^3^ as a touch point between SD and AR is related to the production of research results through experimentation.

 AR is indeed based on the implementation of changes within an organization (or a social context) to tackle a real problem. This takes the form of experimentation in which researchers and practitioners evaluate the proposed changes once implemented in order to produce new knowledge about the system analysed.

 SD is also experimentation friendly as it allows to simulate the behaviour of the modelled system under different conditions. Simulation is a valuable tool to experiment and discover how complex social systems work and where high leverage points may lie.^8^ As reported by Holmström et al,^3^ during the AR process, “the simulation model and user interface were adapted to allow for the testing of a multitude of suggestions and clarified what could work in reality.” In this sense, SD provides a valuable contribution to AR since it allows one to experiment with the social system – eg, the healthcare organization – before implementing costly changes (in terms of money and effort).

###  Collaboration and Engagement

 The third touch point refers to the orientation toward the involvement and engagement of multiple actors and stakeholders that both AR and SD share. As previously mentioned, healthcare systems and organizations’ complexity derives, among other factors, from pluralism and institutional fragmentation. Healthcare services are indeed delivered through the collaboration of different professionals and organizations that may have different values, interests and objectives.^14^ As such, shared understanding on problems and agreement on solutions should not be taken for granted.

 Both AR and SD consider the engagement of the relevant stakeholders as a key moment of the research process. In fact, AR is a research strategy “that is based on actively engaging participants that are willing to share their own perspective on a problem, to collaborate so as to find a mutually accepted solution on a problem.”^3^ On the other hand, an important branch of application of SD is aimed to define the shared mental model of a group of people with respect to a dynamic problem and to translate this into a simulation model.^15^ This is called Group Model Building.

 Even in this case, thus, SD supports and facilitates AR processes since it fosters the definition of shared mental models related to the problems to be addressed and makes these explicit.

## Conclusion

 The analysis of the three touch points emerging from the article of Holmström and colleagues^3^ allowed us to better explore the advantages that integrating SD in AR may bring when doing research in the healthcare domain.

 The integration of SD into AR is allowed by interesting synergies. First of all, the AR strategy provides SD with an important epistemological support. SD has been criticized by researchers producing knowledge following positivism and neo-positivism paradigms with regards to SD models’ ability to mimic reality, their validation, and the use of prediction. Using SD within AR processes may strengthen the positioning of SD as a technique that supports (though non-exclusively) research belonging to the pragmatism philosophy realm.

 On the other hand, SD provides AR with two key features that may improve the production of knowledge in terms of efficiency and effectiveness. The first one is related to simulation. SD simulation allows us to experiment with complex social systems (such as healthcare systems and organizations) and to test solutions and changes before their actual implementation. This fosters learning processes, as multiple solutions can be easily and rapidly tested, saving also effort in terms of time and money as only well-performing changes tested through simulation would be implemented.

 The second one refers to the systematization of the knowledge emerging from the multiple mental models of the people involved in the action and the elicitation of the shared mental model through Group Model Building. As such, SD results being a valuable tool to improve the collaboration within the group of stakeholders called to contribute to the action. Moreover, the elicitation of the shared mental model may better clarify and represent the contribution to knowledge produced by the AR process.

## Ethical issues

 Not applicable.

## Competing interests

 Author declares that he has no competing interests.

## Author’s contribution

 GN is the single author of the paper.
